# MitoQ Triggers Mitochondrial Collapse and Apoptotic Death in Glioblastoma Associated with KATP Channel Expression Changes

**DOI:** 10.1007/s11064-026-04742-6

**Published:** 2026-03-30

**Authors:** Alp Karaaslan, Ceyhan Hacioglu

**Affiliations:** 1 Department of Neurosurgery, İstanbul Şehit Prof. Dr. İlhan Varank Sancaktepe Training and Research Hospital, İstanbul, Turkey; 2 Department of Medical Biochemistry, Faculty of Medicine, Düzce University, Düzce, Turkey

**Keywords:** Glioblastoma, Mitochondrial dysfunction, ATP-sensitive potassium (KATP) channels, Mitoquinone (MitoQ)

## Abstract

Glioblastoma (GBM) is the most aggressive primary brain tumor and remains refractory to current therapies due to its pronounced metabolic heterogeneity and mitochondrial adaptability. Ion channels, particularly ATP-sensitive-potassium (KATP) channels, have emerged as critical regulators of cellular energy sensing in cancer. This study evaluated the mitochondrial-targeted agent MitoQ in GBM and explored its potential association with KATP channels. In this study, the cytotoxic potential of MitoQ was systematically evaluated in three genetically distinct GBM cell lines. Cell viability was assessed using concentration–response analyses to identify differential sensitivity. Baseline expression of KATP-channel components (KCNJ11/Kir6.2, ABCC8/SUR1, and CCDC51) was quantified by qRT-PCR and Western blotting. Mechanistic analyses were subsequently performed in the most sensitive cell line and included mitochondrial ROS measurement (MitoSOX), confocal assessment of mitochondrial morphology, Seahorse XF-based bioenergetic profiling, ATP/ADP ratio quantification, analysis of autophagic flux via LC3-II/p62 turnover with bafilomycin-A1, and caspase-3/7-based apoptosis detection. U87 cells exhibited the lowest IC₅₀ for MitoQ and showed significantly higher baseline expression of KATP channel subunits compared to U251 and T98G cells. Acute MitoQ exposure (10 µM, 6 h) in U87 cells induced marked mitochondrial superoxide accumulation, extensive mitochondrial fragmentation, severe suppression of oxidative phosphorylation, and ATP depletion. These effects were associated with selective downregulation of Kir6.2 and the mitochondrial KATP-associated component CCDC51, impaired autophagic flux with p62 accumulation, and robust activation of executioner caspases. In conclusion, MitoQ may induce mitochondrial dysfunction in metabolically primed GBM cells, and cellular sensitivity appears to correlate with a distinct KATP channel expression signature.

**Supplementary Information:**

The online version contains supplementary material available at 10.1007/s11064-026-04742-6.

## Introduction

Potassium channels represent one of the most diverse ion channel families, playing critical roles in regulating membrane potential, cellular signaling, and metabolic homeostasis in both excitable and non-excitable cells [1]. Among these, ATP-sensitive potassium (KATP) channels function as unique metabolic sensors, directly coupling intracellular energetic status to membrane excitability and organellar function [2]. Structurally composed of a pore-forming inwardly rectifying potassium subunit (Kir6.1 or Kir6.2) and a regulatory sulfonylurea receptor (SUR1 or SUR2), these channels close under high ATP conditions and open when intracellular ATP levels decrease, thereby linking cellular metabolism to potassium efflux [3]. While their role in pancreatic beta-cells and cardiomyocytes is well-established, emerging evidence implicates KATP channels in tumor biology, including the regulation of cancer cell proliferation, survival, and response to metabolic stress [4, 5]. In glioblastoma (GBM), the most aggressive primary brain tumor, cellular metabolism is profoundly reprogrammed to support rapid growth, invasion, and therapeutic resistance [6]. The potential involvement of KATP channels in modulating GBM bioenergetics, cell proliferation [7], and resilience to stress presents a compelling yet underexplored avenue for therapeutic intervention.

GBM is characterized by extensive genetic heterogeneity, infiltrative growth, and a notoriously poor prognosis, with median survival rarely exceeding 15 months despite maximal therapy involving surgical resection, temozolomide (TMZ), and radiotherapy [8]. This therapeutic failure is driven by both intrinsic and adaptive resistance mechanisms, including metabolic plasticity, robust DNA repair, potent antioxidant defense systems, and dynamic mitochondrial adaptability [9]. Mitochondrial dysfunction is a hallmark of GBM, not as a mere bystander but as an active contributor to its pathogenesis [10]. Tumors exhibit rewired oxidative phosphorylation, altered mitochondrial dynamics, and dysregulated mitochondrial quality control mechanisms to sustain survival under hypoxia and nutrient deprivation [11]. This metabolic reprogramming creates unique vulnerabilities that are not targeted by conventional therapies. This profound heterogeneity is recapitulated in established preclinical models, which display a wide spectrum of metabolic and resistant phenotypes mirroring the clinical disease [12]. These models range from those with adaptable metabolic pathways to highly proliferative subtypes with enhanced antioxidant defenses, and others characterized by intrinsic chemoresistance and marked mitochondrial robustness [13]. Such phenotypic diversity highlights a central challenge in GBM therapy: identifying and exploiting fundamental mitochondrial vulnerabilities that are conserved across this varied landscape, thereby offering a strategy to overcome adaptive resistance mechanisms.

In this context, mitochondrial-targeted compounds have gained significant interest as potential adjuvants to disrupt tumor bioenergetics and overcome therapy resistance. Mitoquinone (MitoQ), a mitochondria-targeted derivative of the antioxidant coenzyme Q10, has emerged as a promising agent with a dual role [14]. It consists of a ubiquinone moiety linked to a lipophilic triphenylphosphonium cation, which drives its accumulation within mitochondria. At low concentrations, MitoQ acts as a potent antioxidant, scavenging mitochondrial reactive oxygen species (ROS) and protecting against oxidative damage—a property explored in neurodegenerative and cardiovascular diseases [15]. Paradoxically, at higher concentrations or in specific cellular contexts, particularly in cancer cells with already stressed mitochondrial networks, MitoQ can exhibit pro-oxidant effects [16]. It can disrupt the electron transport chain, promote excessive ROS generation, induce mitochondrial membrane depolarization, and trigger apoptotic signaling [17]. This capacity to shift from a protective to a disruptive agent based on dosage and cellular redox state makes it an intriguing candidate for cancer therapy. Preliminary studies in models of breast, colon, and hepatic cancers have shown that MitoQ can inhibit proliferation and sensitize cells to chemotherapy [18–20]. However, its application in GBM remains strikingly limited. A critical gap remains in understanding how MitoQ influences the distinct mitochondrial landscapes of different GBM subtypes, and whether its effects are mediated through specific pathways such as autophagy inhibition, metabolic collapse, or the induction of lethal oxidative stress. Furthermore, the potential interplay between MitoQ’s action and key metabolic regulators like KATP channels, which are known to modulate mitochondrial membrane potential and cellular stress responses in other tissues, has not been investigated in GBM.

Building on the rationale that targeting mitochondrial metabolism represents a viable strategy to overcome therapeutic resistance in GBM, this study aims to systematically investigate the cytotoxic potential of the mitochondria-targeted agent MitoQ across three genetically distinct GBM cell lines: U87, U251, and T98G. We hypothesized that differential baseline mitochondrial phenotypes and stress resilience would dictate variable cellular sensitivity to MitoQ. To address this, a two-phase comparative experimental design was employed. In the first phase, the relative sensitivity of each cell line to MitoQ was determined through comprehensive viability screening, enabling the identification of the most responsive model. In the second phase, in-depth mechanistic analyses were conducted in the selected sensitive cell line to delineate the sequence of mitochondrial events induced by MitoQ exposure, including alterations in ROS production, disruption of autophagic flux, and changes in cellular ATP levels. Furthermore, given the emerging role of KATP channels as metabolic stress sensors in cancer biology, this study preliminarily examined whether the expression and modulation of KATP channel components, particularly the pore-forming subunit Kir6.2, correlates with cellular susceptibility to MitoQ. Through this approach, this study aimed to explore a potential relationship between classical metabolic sensing mechanisms and mitochondrial-targeted therapeutic stress in GBM cells. Notably, our experimental strategy was deliberately designed as a two‑stage process: the first stage employed viability screening across three phenotypically distinct GBM lines to identify the most MitoQ‑sensitive model; the second stage then focused exclusively on that sensitive line to perform an in‑depth, resource‑intensive dissection of the underlying mitochondrial mechanisms.

## Materials and Methods

### Cell Culture

Human GBM cell lines U87, U251, and T98G were obtained from the American Type Culture Collection (ATCC). All cell lines were cultured in Dulbecco’s Modified Eagle’s Medium (DMEM, high glucose, Gibco, 11965092) supplemented with 10% fetal bovine serum (FBS, Gibco, 10270106) and 1% penicillin-streptomycin (Gibco, 15140122). Cultures were maintained in a humidified incubator at 37 °C with 5% CO₂. Cells were routinely passaged at 80–90% confluence.

### Assessment of MitoQ Cytotoxicity

Cell viability in response to MitoQ (MedChemExpress, HY-100116) was assessed using complementary colorimetric assays. Cells were seeded in 96-well plates and treated with a logarithmic dilution series of MitoQ (0.5–80 µM) for 24 h [21].

The Cell Counting Kit-8 (BMU106-EN) was used according to the manufacturer’s instructions. Absorbance was measured at 450 nm. The CCK-8 assay primarily reflects metabolic competence and may underestimate actual survival following mitochondrial perturbation. Therefore, we interpret these results as a metabolic sensitivity index rather than a strict measure of cell death. Engagement of apoptotic pathways was independently validated using caspase-3/7 activation assays.

MitoQ was dissolved in dimethyl sulfoxide (DMSO) to prepare a 20 mM stock solution, which was stored at −20 °C. For all experiments, working concentrations were prepared by diluting the stock in culture medium immediately before use. The final concentration of DMSO in all treatments, including vehicle controls, did not exceed 0.05% (v/v).

### Quantitative Real-Time PCR (qRT-PCR)

Total RNA was extracted using the RNeasy Plus Mini Kit (Qiagen, 74134). cDNA was synthesized from 1 µg RNA using the cDNA Reverse Transcription Kit (Applied Biosystems, 4368814). qRT-PCR was performed in triplicate using SYBR Green Master Mix (Applied Biosystems, A25742) on a QuantStudio 5 Real-Time PCR System. Primers were designed for KCNJ11 (Kir6.2), ABCC8 (SUR1), and CCDC51. GAPDH was used as the endogenous control. Relative gene expression was calculated using the 2^−ΔΔCt^ method. All procedures followed the manufacturer’s protocols. KCNJ11 (Kir6.2): Forward 5′-ACGGTGGTCATCATCGTGGAGC-3′, Reverse 5′-GCCATGGATGATGCACCACAGG-3′; ABCC8 (SUR1): Forward 5′-GCAGGCAGAAGGTGTTGGCCA-3′, Reverse 5′- CGGTAGGTGATGGTGAGGTTCGG-3′; CCDC51: Forward 5′-CGGCAGTAGACGCTGGACGC-3′, Reverse 5′-CCGGGACATGACCTCCAGGG-3′; GAPDH: Forward 5′-GCTAAGGTGAAGGTCGGAGTGCC-3′, Reverse 5′-GCTAAGATGGTGATGGGATGGTC-3′.

### Western Blotting

Whole-cell protein lysates were prepared using RIPA buffer containing protease inhibitors. Protein concentration was determined via the BCA assay (Pierce, 23225). Equal amounts of protein (20 µg) were separated on 10–12% SDS-PAGE gels and transferred to PVDF membranes. Membranes were blocked and probed overnight at 4 °C with primary antibodies against Kir6.2 (PA5-99440), SUR1 (PA5-103639), CCDC51 (PA5-52767), LC3-II (PA1-46286), p62 (PA5-20839), and GAPDH (5–35235). HRP-conjugated secondary antibodies and enhanced chemiluminescence substrate (Bio-Rad, 1705060) were used for detection. Densitometric analysis was performed using ImageJ software.

### Immunofluorescence and Confocal Microscopy

Cells were plated on glass coverslips, fixed with 4% paraformaldehyde, and permeabilized with 0.1% Triton X-100. After blocking, cells were labeled with MitoTracker Deep Red FM (Invitrogen, M22426). Images were acquired using a Zeiss LSM 880 confocal microscope with 40x magnification.

For each experimental condition, at least 50 cells from seven independent experiments (total ≥ 350 cells per condition) were randomly selected and analyzed in a blinded manner regarding the treatment groups. Image analysis was performed using ImageJ software with the MiNa plugin. Briefly, maximum intensity projections were generated, and a uniform threshold was applied to create binary masks of mitochondrial signals. The MiNa plugin was then used to quantify key parameters including mean mitochondrial length (µm), branch count per mitochondrion, and a mitochondrial network integrity index (a composite metric inversely related to fragmentation). Cells exhibiting over-confluence or poor-quality staining were excluded from the analysis.

### Mitochondrial ROS Production

Mitochondrial superoxide was detected using MitoSOX Red (Invitrogen, M36008). Cells were incubated with 5 µM MitoSOX for 20 min at 37 °C, washed, and fluorescence was quantified immediately by fluorescence microscopy. Image acquisition was performed using a Zeiss Axio Observer 7 microscope equipped with a 40x objective with identical exposure settings across all conditions. For each independent experiment (*n* = 7), ≥ 50 cells per condition were randomly selected and analysed in a blinded manner using ImageJ software. Mean fluorescence intensity was measured after background subtraction and normalised to the number of cells per field. This approach provides single‑cell resolution and permits detection of heterogeneous responses, which is not achievable with bulk fluorometric assays.

### Seahorse XF Analysis and ATP/ADP Ratio Quantification

Mitochondrial respiratory function was evaluated using the Seahorse XF extracellular flux analyzer (Agilent Tech.) via a standard Mito Stress Test. Cells were seeded into Seahorse XF96 microplates at a density of 1 × 10^4^ cells per well and allowed to adhere overnight. This density was determined through prior optimization to ensure a linear response in oxygen consumption rate. Cells were then treated with MitoQ for 6 h at a concentration below the 24-h IC₅₀ to assess early bioenergetic alterations. Prior to measurement, culture medium was replaced with Seahorse XF assay medium supplemented with glucose (10 mM), pyruvate (1 mM), and L-glutamine (2 mM), and equilibrated in a non-CO₂ incubator. Oxygen consumption rate (OCR) was recorded at baseline and following sequential injections of oligomycin (1 µM), FCCP (1.5 µM), and rotenone/antimycin A (0.5 µM each) to determine basal respiration, ATP-linked respiration, maximal respiration, and spare respiratory capacity.

Intracellular ATP and ADP levels were measured using the Luminescent ATP/ADP Ratio Assay Kit (Abcam, ab65313). Luminescence was recorded, and ratios were calculated as per the manufacturer’s instructions.

### Caspase-3/7 Activity Analysis

Caspase-3/7 activation was evaluated using the Muse^®^ Caspase-3/7 Assay Kit (MCH100108), which detects effector caspases engaged during the execution phase of apoptosis. To concurrently assess plasma membrane integrity, 7-aminoactinomycin D (7-AAD) was incorporated as an exclusion dye, allowing discrimination between early apoptotic, late apoptotic, and non-viable cell populations. For each condition, cells were treated with MitoQ for 6 h, harvested by gentle trypsinization, and washed twice with cold PBS. Cell pellets were resuspended in assay buffer and incubated with 5 µL of the Caspase-3/7 reagent for 30 min at 37 °C, enabling intracellular binding to the activated caspase-3/7 cleavage products. Following this incubation, 150 µL of 7-AAD was added to each sample, mixed thoroughly, and immediately subjected to single-cell fluorescence analysis using the Muse^®^ Cell Analyzer.

### Statistical Analysis

All quantitative data are presented as mean ± standard error of the mean (SEM) derived from at least seven independent biological replicates. Statistical analyses were performed using GraphPad Prism software (version 8.0; GraphPad). Prior to inferential testing, data distributions were assessed for normality using the Shapiro–Wilk test to ensure compliance with parametric test assumptions. For comparisons involving more than two experimental groups, one-way or two-way analysis of variance (ANOVA) was applied as appropriate, followed by Tukey’s post hoc multiple comparisons test to identify specific group differences. For pairwise comparisons between two groups, unpaired two-tailed Student’s t-tests were used. Nonlinear regression analysis was applied to concentration–response curves obtained from CCK-8 viability assays using a four-parameter logistic model to calculate half-maximal inhibitory concentration (IC₅₀) values. *P*-value < 0.05 was considered statistically significant. Statistical significance is denoted in figures as follows: *p* < 0.05 (*), *p* < 0.01 (**), *p* < 0.001 (***), and *p* < 0.0001 (****).

## Results

### Baseline KATP Subunit Expression Reveals Heterogeneity Across GBM Cell Lines

**Fig. 1:**
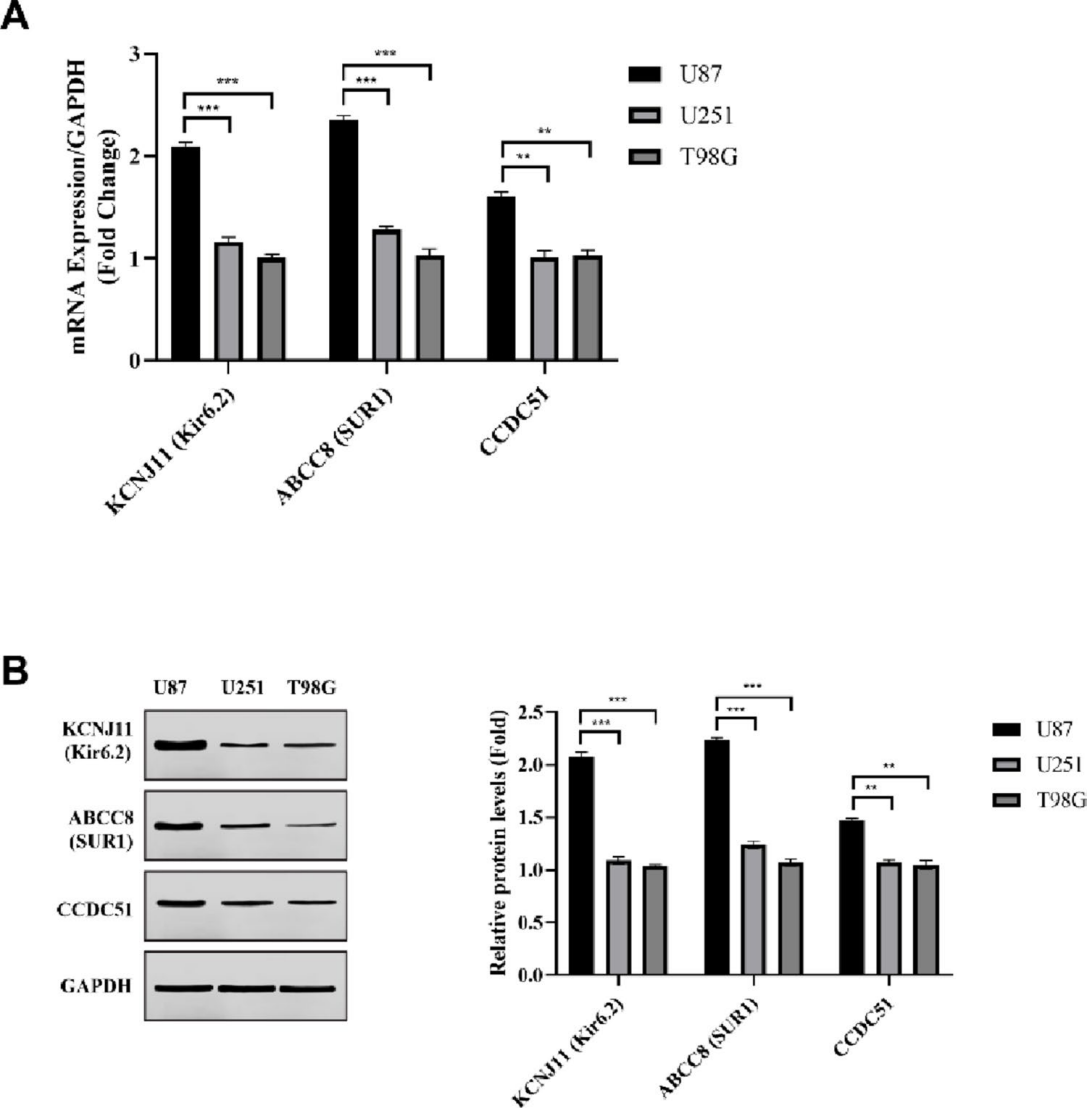
Expression profiling of KATP channel subunits reveals heterogeneity across GBM cell lines. **A** qRT-PCR analysis of plasmalemmal KATP channel subunits (KCNJ11 and ABCC8) and mitochondrial-associated KATP component CCDC51 in U87, U251, and T98G cells. Gene expression levels were normalized to GAPDH. **B** Representative Western blots illustrating protein expression of Kir6.2, SUR1, and CCDC51 across GBM cell lines, with GAPDH used as a loading control. Densitometric quantification of Western blot signals normalized to GAPDH. Data were presented as mean ± SEM from seven independent experiments. Statistical significance was determined using two-way ANOVA. **p < 0.01, ***p < 0.001

To establish a potential molecular basis for differential metabolic responses, we first characterized the baseline expression landscape of key KATP channel components across the three GBM cell lines. Integrated analysis using qPCR and Western blotting collectively revealed a marked heterogeneity in the expression of both plasmalemmal and mitochondrial-associated subunits (Fig. 1).

Transcriptional profiling via qPCR corroborated and expanded upon the protein expression hierarchy, revealing significant percentage increases in mRNA levels for all KATP components in U87 cells (Fig. 1A). For the primary pore-forming subunit, KCNJ11 (Kir6.2), transcript levels were about 115% greater in U87 versus T98G cells (*p* < 0.001). This pattern extended to the regulatory subunits, where ABCC8 (SUR1) mRNA was 133% more abundant in U87 relative to T98G (*p* < 0.001). Finally, transcript levels for the mitochondrial component CCDC51 were about 57% greater in U87 cells than in U251 cells (*p* < 0.01). This consistent transcriptional gradient suggests that the differential protein expression of KATP channel infrastructure across these GBM lines is fundamentally regulated at the mRNA level.

Quantification of protein expression confirmed these pronounced disparities across all major KATP channel components. Densitometric analysis of Western blots established a clear expression hierarchy, with U87 cells consistently exhibiting the highest protein levels (Fig. 1B). The difference in the primary pore-forming subunit, Kir6.2 (KCNJ11), was even more striking, with U87 cells showing about a 2.1-fold increase over T98G cells (*p* < 0.001). This pattern extended to the regulatory sulfonylurea receptors: SUR1 (ABCC8) was found to be roughly 2.3-fold more abundant in U87 versus T98G cells (*p* < 0.001). Finally, the mitochondrial-associated component CCDC51 was about 1.4-fold more prevalent in U87 cells than in U251 cells (*p* < 0.01). This comprehensive gradient—from U87 (highest) to U251 (moderate) to T98G (lowest)—suggests an intrinsically more robust and potentially more complex KATP channel infrastructure in the U87 line, encompassing both plasmalemmal and mitochondrial-associated components.

### MitoQ Viability Screening Identifies U87 as the Most Sensitive GBM Line

**Fig. 2:**
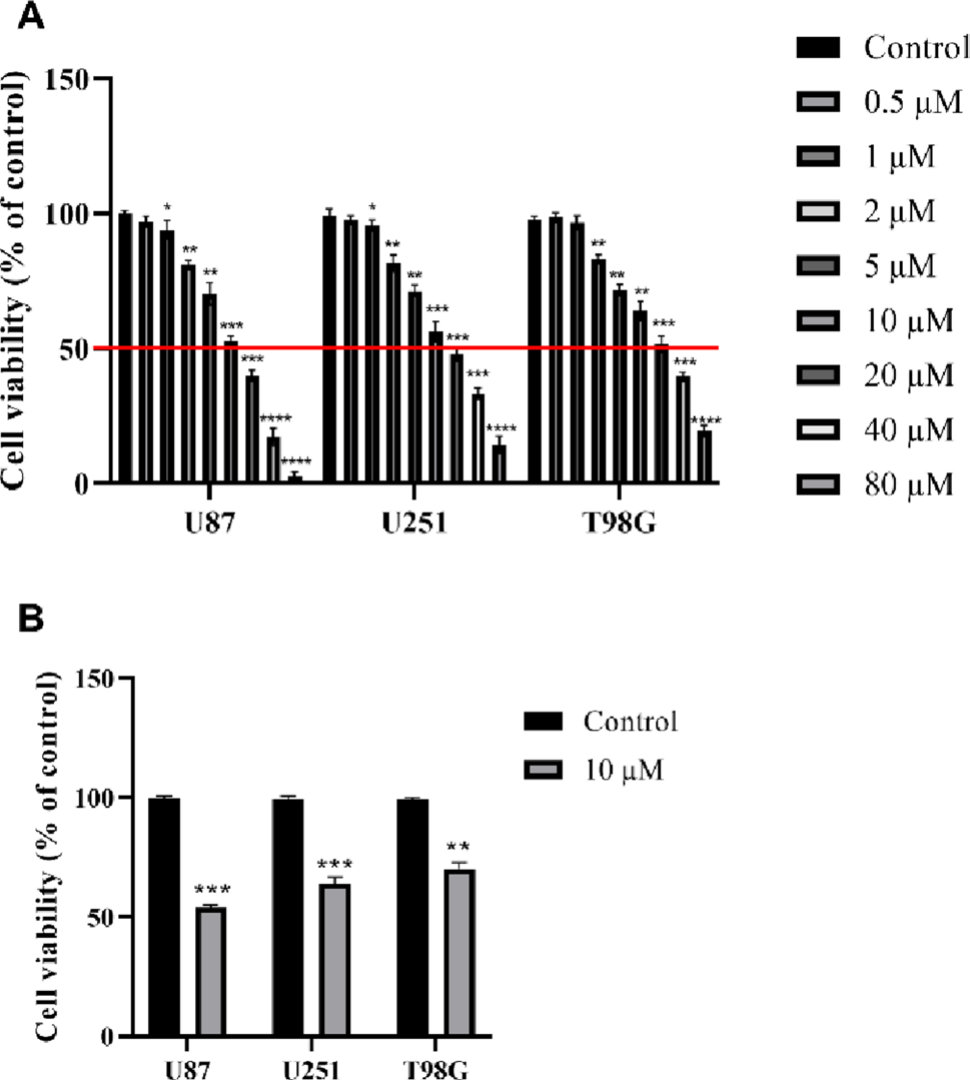
MitoQ exhibits differential antiproliferative activity across GBM cell lines. U87, U251, and T98G glioblastoma cells were treated with increasing concentrations of MitoQ (0.5–80 µM) for 24 h, and cell viability was assessed using the CCK-8 assay. **A** Concentration–response curves reveal a pronounced leftward shift in U87 cells, indicative of increased sensitivity to mitochondrial targeting. **B** Representative viability measurements at a sub-IC₅₀ dose (10 µM) highlight differential cytotoxic responses among cell lines. Data were presented as mean ± SEM from at least seven independent experiments. Statistical significance was determined using two-way ANOVA and unpaired Student’s *t*-tests. *p < 0.05, **p < 0.01, p < ***0.001, ****p < 0.0001

The antiproliferative efficacy of the mitochondrial-targeting agent MitoQ was systematically evaluated across three genetically distinct GBM cell lines—U87, U251, and T98G—via comprehensive concentration-response analysis (Fig. 2). A 24-h treatment with MitoQ (0.5–80 µM) revealed differential sensitivity among the lines, as measured by CCK-8 viability assays. U87 cells exhibited the highest susceptibility, with an estimated half-maximal inhibitory concentration (IC₅₀) of approximately 11.4 µM. In contrast, U251 cells demonstrated moderate sensitivity with an IC₅₀ of 18.6 µM, while T98G cells were the most resistant, requiring an IC₅₀ of about 25.5 µM for a similar cytotoxic effect (Fig. 2A; for detailed IC₅₀ values with confidence intervals see Supplementary Table S1, and for dose-response curve modeling see Supplementary Figure S1).

Based on this clear differential sensitivity profile, U87 cells were selected for all subsequent in-depth mechanistic investigations. This selection is consistent with our pre‑defined two‑phase strategy, which prioritises comprehensive mechanistic elucidation in the most responsive cellular context. We recognise that the mechanisms operating in the less sensitive U251 and T98G lines may differ, and this important question is left for future investigation. This choice was further substantiated by representative CCK-8 viability readouts obtained at a sub-IC₅₀ concentration of MitoQ (10 µM), which revealed a pronounced and statistically significant reduction in cell viability in U87 cells following 24 h exposure (Fig. 2B). At this concentration, U87 cells exhibited a viability of 53 ± 1.4%, corresponding to a 47% reduction relative to controls, whereas the same treatment resulted in only moderate viability decreases in U251 cells (61 ± 5.2%; 39% reduction) and minimal effects in the more resistant T98G cells (68 ± 3.5%; 32% reduction). Consistent with the graded IC₅₀ values across the three GBM lines (U87: 11.4 ± 0.9 µM; U251: 18.6 ± 1.4 µM; T98G: 25.5 ± 1.8 µM), these data confirm that a concentration below the 24-h IC₅₀ (10 µM) was used for U87 cells while remaining well below the cytotoxic threshold for the less sensitive lines.

### MitoQ Modulates KATP Channel Subunit Expression in U87 Cells

**Fig. 3:**
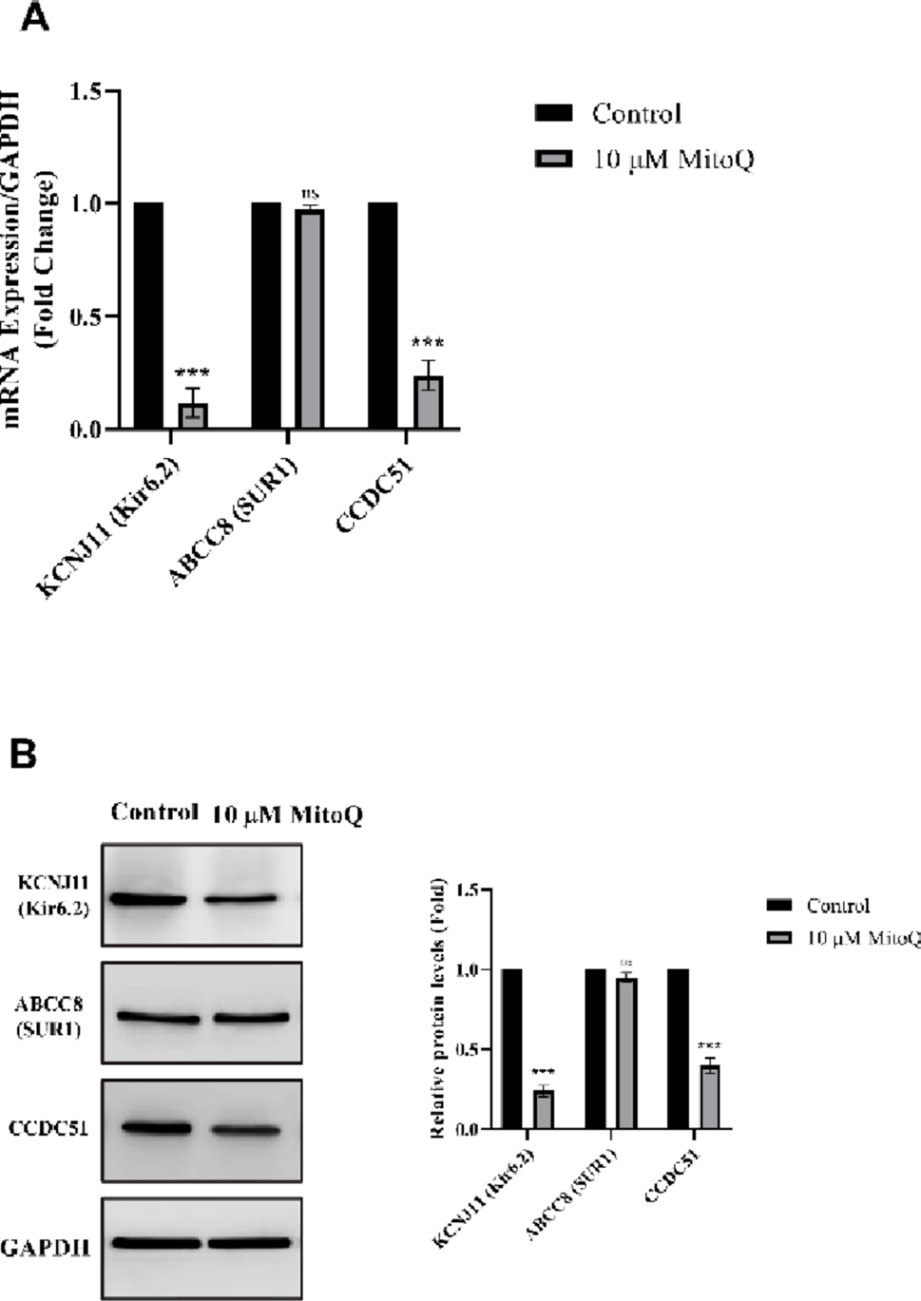
MitoQ selectively downregulates pore-forming and mitochondrial-associated KATP channel subunits in U87 cells. **A** Quantitative RT-PCR analysis of KATP channel subunit transcripts in U87 cells following treatment with MitoQ (10 µM, 6 h). Transcript levels were normalized to GAPDH and expressed relative to controls. Transcript levels were normalized to GAPDH and expressed as fold-change relative to the vehicle-treated control group (set to 1). **B** Representative Western blot images showing protein expression of Kir6.2, SUR1, and CCDC51, with GAPDH as a loading control. Data were presented as mean ± SEM from seven independent experiments. Statistical significance was determined using unpaired Student’s *t*-test. *** *p* < 0.001; ns, not significant

Given the pronounced enrichment of both plasmalemmal and mitochondrial KATP channel components in U87 cells at baseline, we next examined whether acute mitochondrial stress induced by MitoQ alters the expression profile of these metabolic sensors (Fig. 3). U87 cells were treated with MitoQ at a sub-IC₅₀ concentration (10 µM) for 6 h, a time point selected to capture early transcriptional and translational responses preceding overt cell death.

Quantitative RT-PCR analysis revealed a selective and differential regulation of KATP subunit transcripts following MitoQ exposure (Fig. 3A). Expression of the pore-forming subunit KCNJ11 (Kir6.2) was significantly reduced, exhibiting an approximate 48% decrease relative to controls (*p* < 0.001). In contrast, mRNA levels of the regulatory subunit ABCC8 (SUR1) showed only modest, non-significant changes following acute MitoQ treatment, indicating a preferential sensitivity of the pore-forming components to mitochondrial stress (*p* > 0.05). Notably, expression of the mitochondrial-associated KATP component CCDC51 was significantly suppressed, demonstrating an approximate 41% reduction compared to untreated cells (*p* < 0.001). Consistent with the transcriptional data, Western blot analysis confirmed a corresponding decrease at the protein level (Fig. 3B). Densitometric quantification revealed that Kir6.2 protein abundance was reduced by 52% following MitoQ treatment (*p* < 0.001), whereas changes in SUR1 protein expression did not reach statistical significance. Importantly, CCDC51 protein expression was reduced by 43% in MitoQ-treated U87 cells (*p* < 0.001), paralleling its transcriptional downregulation.

Collectively, these data suggest that acute mitochondrial disruption by MitoQ selectively suppresses the expression of pore-forming and mitochondrial-associated KATP channel components in U87 cells, while largely sparing regulatory SUR subunits. This pattern suggests that MitoQ-induced mitochondrial stress is associated with reduced expression of specific KATP channel components that participate in mitochondrial bioenergetic sensing.

### MitoQ Induces ROS Accumulation in U87 Cells

**Fig. 4:**
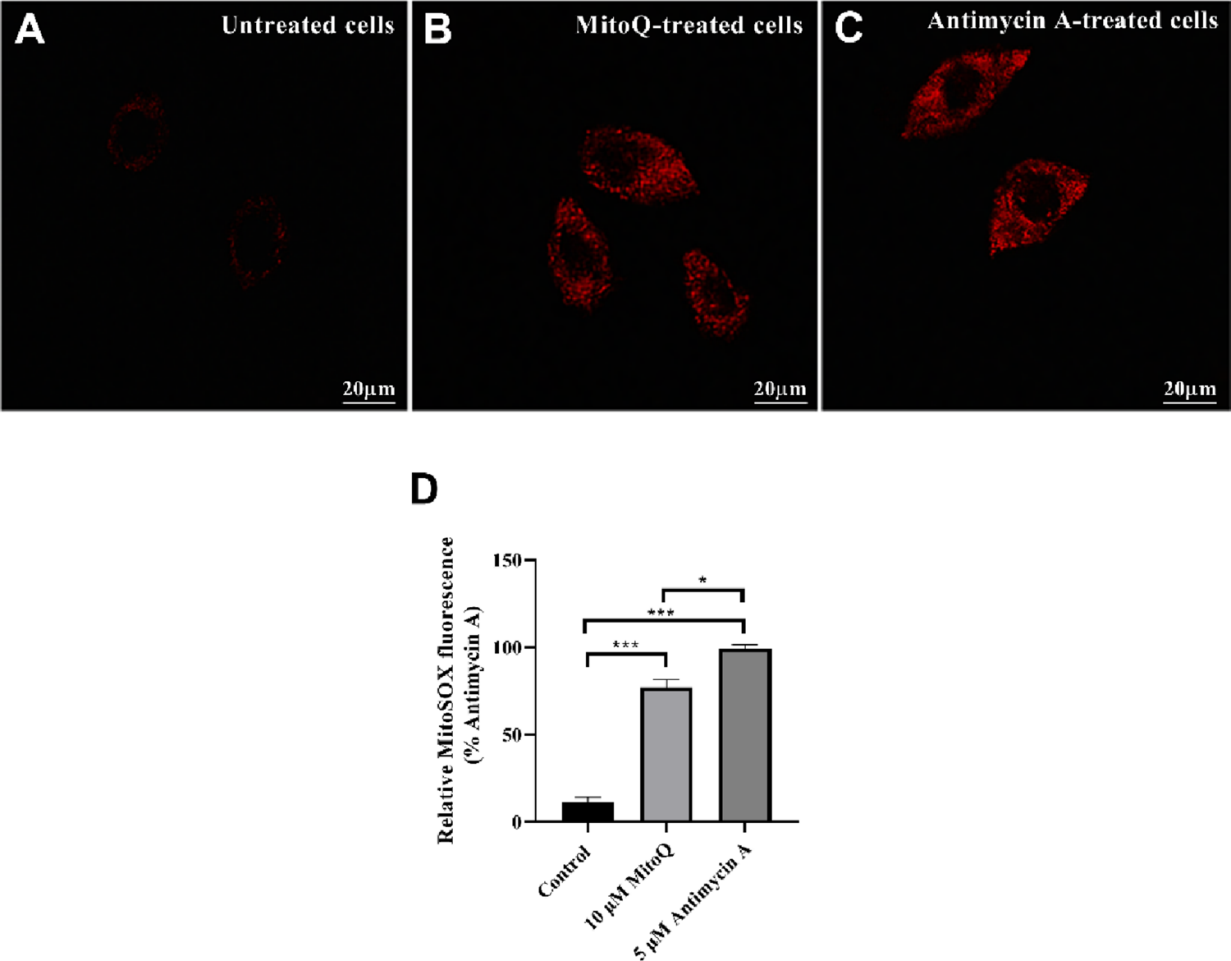
MitoQ triggers acute mitochondrial oxidative stress in U87 cells. U87 cells were exposed to MitoQ (10 µM, 6 h), or Antimycin A (5 µM, 2 h). **A** Representative confocal images of MitoSOX Red staining reveal minimal basal mitochondrial superoxide in untreated cells (**B**), whereas MitoQ induces a marked increase in mitochondrial ROS, approaching levels observed with Antimycin A (**C**). Quantitative analysis demonstrates a significant elevation of MitoSOX fluorescence following MitoQ treatment (**D**). Scale bar = 20 μm. Data were presented as mean ± SEM from seven independent experiments. Statistical significance was assessed using one-way ANOVA followed by Tukey’s multiple comparisons test. *p < 0.05, ***p < 0.001

To determine whether the pronounced cytotoxicity of MitoQ in U87 cells was driven by acute mitochondrial oxidative stress, we next examined its effects on mitochondrial ROS generation under early, sublethal conditions (Fig. 4). U87 cells were subjected to a short-term 6 h exposure to MitoQ in order to capture primary mitochondrial perturbations preceding irreversible cell death. Antimycin A (5 µM, 2 h) was employed as a reference compound because it is a well-established inhibitor of mitochondrial complex III, known to promote robust superoxide generation at the Q site by interrupting electron transfer between cytochrome b and cytochrome c. Thus, Antimycin A serves as a benchmark for maximal mitochondrial ROS production arising from electron transport chain dysfunction.

Untreated U87 cells exhibited low basal MitoSOX fluorescence, consistent with intact redox homeostasis (Fig. 4A). In contrast, MitoQ treatment led to a 3.2-fold increase in mitochondrial superoxide levels compared to untreated controls (p < 0.001), reflecting substantial mitochondrial oxidative stress (Fig. 4B). Antimycin A, a canonical complex III inhibitor, elicited a 4.1-fold increase relative to control (Fig. 4C). When expressed relative to the Antimycin A response, MitoQ reached approximately 77% of the maximal ROS induction (p < 0.05 vs. Antimycin A; Fig. 4D).”

### MitoQ Causes Severe Mitochondrial Fragmentation and Loss of Bioenergetic Capacity in U87 Cells

**Fig. 5:**
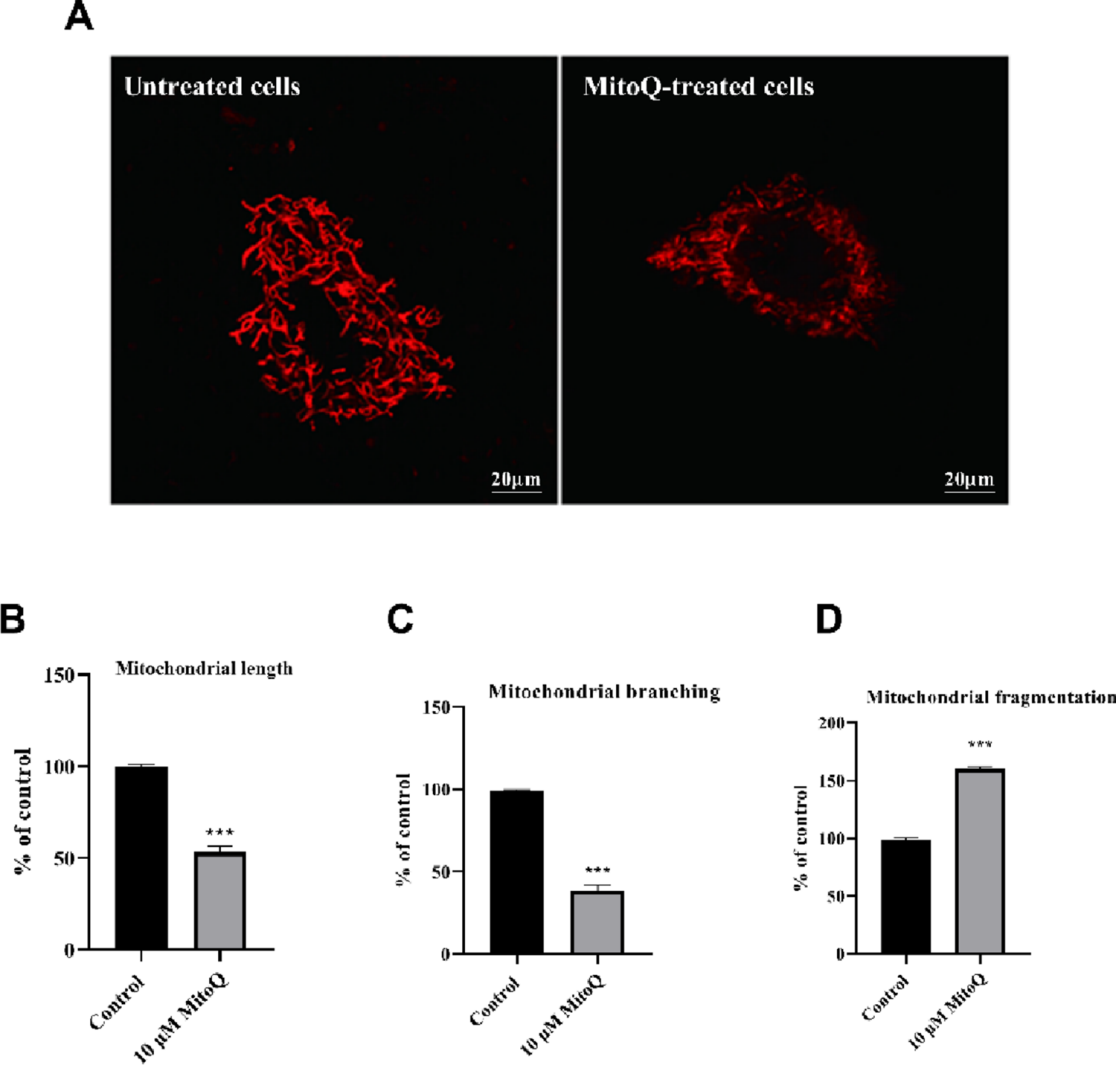
MitoQ rapidly disrupts mitochondrial network architecture in U87 glioblastoma cells. U87 cells were treated with MitoQ (10 µM) for 6 h to induce acute mitochondrial stress. **A** Representative confocal images following MitoTracker Deep Red staining reveal an elongated and interconnected mitochondrial network in untreated cells, whereas MitoQ exposure results in pronounced mitochondrial fragmentation and loss of tubular morphology. Scale bar = 20 μm. Quantitative morphometric analyses demonstrate that MitoQ significantly reduces (**B**) mean mitochondrial length and (**C**) branching index, while markedly increasing (**D**) the mitochondrial fragmentation ratio. All mitochondrial morphometric analyses were performed exclusively on high‑resolution (40x) confocal images, as this magnification is required for accurate quantification using the MiNa plugin. Data were presented as mean ± SEM from seven independent experiments. Statistical significance was assessed using unpaired Student’s t-test. ***p < 0.001

Given the marked increase in mitochondrial ROS and early bioenergetic stress induced by MitoQ, we next investigated whether these alterations translated into structural remodeling of the mitochondrial network. Mitochondrial morphology was analyzed by confocal microscopy following acute exposure to MitoQ (10 µM, 6 h), a time point selected to capture stress-induced network remodeling prior to widespread cell death. Mitochondria were visualized using MitoTracker Deep Red. Untreated U87 cells displayed an elongated, interconnected mitochondrial network characterized by tubular morphology and extensive branching, consistent with a metabolically active and structurally intact mitochondrial population (Fig. 5A). In contrast, MitoQ-treated cells exhibited a striking collapse of mitochondrial architecture. Quantitative analysis of ≥ 350 cells per condition from seven independent experiments revealed that mean mitochondrial length was reduced by approximately 55% compared to controls (*p* < 0.001), indicating a pronounced shift away from elongated mitochondrial structures (Fig. 5B). This shortening was accompanied by 62% reduction in mitochondrial branching index (*p* < 0.001), reflecting a loss of network interconnectivity and suppression of fusion-associated morphology (Fig. 5C).

Consistent with these findings, the mitochondrial fragmentation ratio increased by 59.1% following MitoQ exposure relative to control cells (Fig. 5D; *p* < 0.001). This fragmentation was visually evident as a transition from filamentous mitochondria to punctate, rounded structures distributed throughout the cytoplasm. Mitochondrial fragmentation is not merely a structural epiphenomenon but a direct morphological correlate of functional decline. Elongated mitochondrial networks maintain cristae integrity and optimize electron transport chain supercomplex assembly, whereas fragmented mitochondria exhibit disrupted cristae architecture, reduced respiratory complex activity, and impaired ATP synthesis capacity. Thus, the pronounced shift from tubular to punctate mitochondrial morphology observed in MitoQ-treated U87 cells provides structural evidence underlying the concomitant loss of oxidative phosphorylation and bioenergetic collapse. Importantly, total mitochondrial fluorescence intensity per cell did not differ significantly between groups, indicating that the observed changes were attributable to network remodeling rather than mitochondrial loss.

### MitoQ Profoundly Impairs Mitochondrial Respiratory Function and Cellular Energetic Status in U87 Cells

To determine whether the pronounced mitochondrial fragmentation and oxidative stress induced by MitoQ translated into functional impairment of oxidative phosphorylation, we next assessed key mitochondrial respiratory parameters in U87 cells under acute stress conditions (Table 1). Basal respiration, maximal respiration, ATP-linked oxygen consumption rate (OCR), and spare respiratory capacity were quantified following a 6 h exposure to MitoQ at a concentration below the 24-h IC₅₀ (10 µM), a time point selected to capture primary bioenergetic failure prior to overt cell death.

**Table 1:** MitoQ induces acute mitochondrial respiratory failure and energetic collapse in U87 glioblastoma cells

Parameter	Control	MitoQ	%/Fold Change	*p*-value
Basal respiration (pmol O₂/min)	98.4 ± 6.2	54.1 ± 4.8	↓ 45.0%	*p* < 0.001
ATP-linked OCR (Oligomycin OCR)	62.7 ± 5.1	28.4 ± 3.6	↓ 54.7%	*p* < 0.001
Maximal respiration (FCCP-stimulated)	165.3 ± 9.8	67.9 ± 6.5	↓ 58.9%	*p* < 0.001
Spare respiratory capacity (pmol O₂/min)	66.9 ± 7.2	13.8 ± 2.4	↓ 79.4%	*p* < 0.001
ATP/ADP ratio	1.00 ± 0.07	0.35 ± 0.04	↓ 65.0%	*p* < 0.001

U87 cells were treated with MitoQ (10 µM) for 6 h to capture early mitochondrial dysfunction preceding overt cell death. Mitochondrial respiration was assessed using the Seahorse XF Mito Stress Test. Basal respiration was significantly reduced following MitoQ exposure, indicating suppression of steady-state mitochondrial oxygen consumption. ATP-linked oxygen consumption rate (OCR), calculated as the difference between basal respiration and oligomycin-inhibited OCR, was significantly decreased in MitoQ-treated cells, indicating compromised ATP synthesis. Maximal respiration, measured after FCCP-induced uncoupling, was markedly diminished, reflecting impaired electron transport chain capacity. Spare respiratory capacity, representing the ability of mitochondria to respond to increased energetic demand, was nearly abolished following MitoQ treatment. Cellular energy status was further evaluated by measuring the intracellular ATP/ADP ratio, which showed a pronounced reduction in response to MitoQ. Data were presented as mean ± SEM from seven independent experiments. Statistical significance was determined using unpaired Student’s *t*-test. *p* < 0.001 versus control

Basal respiration was significantly reduced in MitoQ-treated U87 cells compared to controls, exhibiting an approximate 45% decrease (*p* < 0.001), indicating a substantial suppression of steady-state mitochondrial oxygen consumption. This impairment was further exacerbated under conditions of maximal respiratory demand. Maximal respiration, assessed following uncoupling, was diminished by nearly 60% relative to control cells (*p* < 0.001), reflecting a marked loss of electron transport chain capacity and limited ability to respond to increased energetic requirements. Consistent with these findings, ATP-linked OCR—representing the fraction of oxygen consumption directly coupled to ATP synthesis—was reduced by approximately 55% in response to MitoQ treatment (*p* < 0.001). This decline indicates a profound uncoupling between mitochondrial respiration and ATP production, likely arising from combined effects of mitochondrial membrane depolarization, oxidative damage to respiratory complexes, and disrupted cristae architecture. Importantly, the spare respiratory capacity, a key indicator of mitochondrial resilience and metabolic flexibility, was almost completely abolished following MitoQ exposure, showing an approximate 70% reduction compared to untreated cells (*p* < 0.001).

To directly assess the downstream energetic consequences of this respiratory failure, intracellular ATP/ADP ratios were measured as an integrated readout of cellular energy status. Acute MitoQ treatment resulted in a dramatic reduction in the ATP/ADP ratio by approximately 65% relative to untreated controls (*p* < 0.001), confirming severe energetic collapse.

### MitoQ Impairs Autophagic Flux and Leads to Accumulation of Autophagy Markers in U87 Cells

**Fig. 6:**
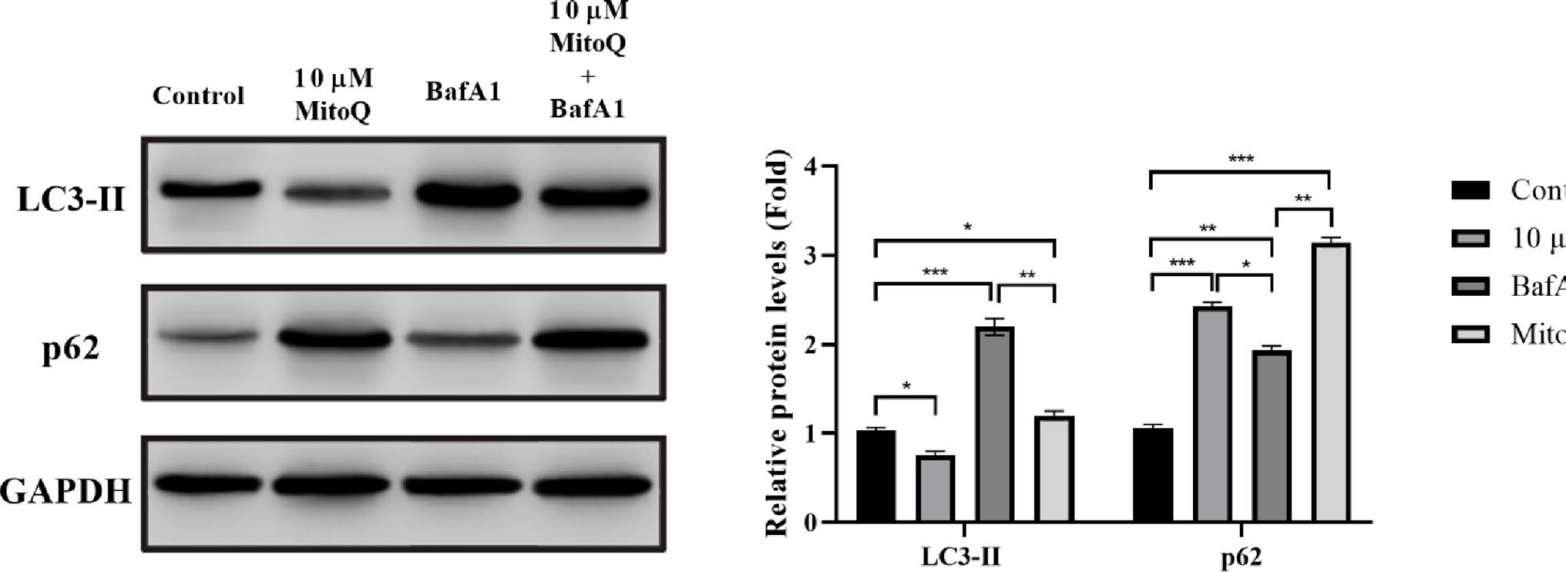
MitoQ impairs autophagy-associated autophagic flux and promotes the accumulation of dysfunctional mitochondria in U87 cells. Autophagy dynamics were evaluated by Western blot analysis of LC3 and p62 in U87 glioblastoma cells subjected to acute mitochondrial stress. Cells were treated with vehicle or MitoQ (10 µM, 6 h), with or without co-treatment with bafilomycin A1 (BafA1; 100 nM, final 2 h). Representative Western blot images showing LC3-II and p62 protein levels in untreated and treated U87 cells. GAPDH used as a loading control. Data were presented as mean ± SEM from seven independent experiments. Statistical significance was determined using one-way ANOVA followed by Tukey’s multiple comparisons test. *p < 0.05, **p < 0.01, p < ***0.001

Autophagic flux, a key cellular clearance pathway activated in response to mitochondrial damage, was assessed in U87 cells by Western blot analysis of the autophagy markers LC3 and p62 under conditions of acute mitochondrial stress (Fig. 6). To specifically evaluate mitophagic flux rather than static autophagosome abundance, bafilomycin A1 (BafA1; 100 nM) was employed. BafA1 is a selective inhibitor of the vacuolar H⁺-ATPase that prevents lysosomal acidification and blocks autophagosome–lysosome fusion, thereby allowing discrimination between increased autophagosome formation and impaired autophagic degradation. Under basal conditions, untreated U87 cells displayed normal autophagic activity, as evidenced by low p62 levels and an accumulation of LC3-II upon BafA1 treatment, consistent with autophagic flux. In contrast, acute exposure to MitoQ markedly disrupted autophagy progression. Western blot analysis revealed a significant attenuation of LC3-II turnover in MitoQ-treated cells, as indicated by an increase in LC3-II levels in the presence of BafA1 compared to controls. Quantitative densitometric analysis demonstrated that LC3-II accumulation in the MitoQ + BafA1 group was reduced by approximately 48% relative to the BafA1 condition (*p* < 0.001), indicating impaired autophagosome processing and reduced mitophagic flux. Concomitantly, MitoQ treatment resulted in a pronounced accumulation of the autophagy adaptor protein p62/SQSTM1, a canonical marker of defective autophagic degradation. Densitometric quantification revealed that p62 protein levels were increased by approximately 2.4-fold in MitoQ-treated U87 cells compared to vehicle controls (*p* < 0.001). Importantly, p62 accumulation was further exacerbated in the presence of BafA1, supporting the conclusion that MitoQ impairs autophagic clearance rather than stimulating compensatory autophagosome formation.

### MitoQ-Triggered Mitochondrial Stress Drives Apoptotic Cell Death

**Fig. 7:**
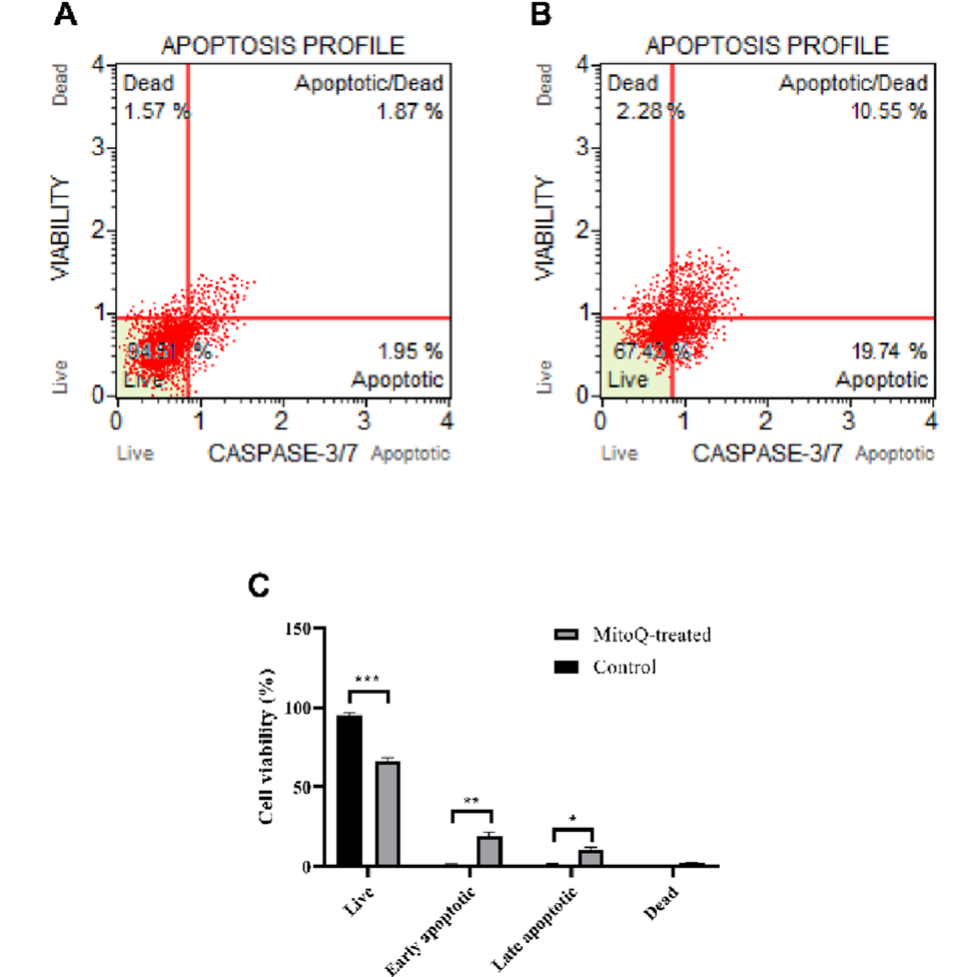
MitoQ-induced mitochondrial stress activates executioner caspases and promotes apoptotic cell death in U87 cells. U87 cells were treated with vehicle MitoQ (10 µM) for 6 h at a concentration below the 24-h IC₅₀. Apoptosis was assessed using the Muse^®^ Caspase-3/7 Assay Kit, which enables single-cell detection of activated caspase-3/7 in combination with 7-AAD staining. Representative dot plots showing the distribution of live, apoptotic, apoptotic/dead, and dead cell populations. **A** Untreated U87 cells, **B** MitoQ-treated U87 cells and (**C**) Quantitative bar graphs summarize the percentages of live, apoptotic, and dead cells

Apoptosis was quantitatively evaluated using the Caspase-3/7 Assay Kit, which detects activated (cleaved) caspase-3/7 in individual cells and enables discrimination between live, apoptotic, apoptotic/dead, and dead. U87 cells were treated with MitoQ at a concentration below the 24-h IC₅₀ (10 µM) for 6 h, corresponding to the acute mitochondrial stress window used in upstream mechanistic analyses. Under basal conditions, vehicle-treated cells exhibited low levels of caspase-3/7 activation, consistent with minimal spontaneous apoptosis (Fig. 7A). Quantitative single-cell analysis revealed a marked increase in caspase-3/7–positive cells following MitoQ treatment. Specifically, the proportion of cells exhibiting apoptotic cells increased by approximately 20% compared to untreated controls (Fig. 7A; *p* < 0.001). Quantification experiments (Fig. 7C) revealed that MitoQ treatment significantly increased the total apoptotic cell population (early + late apoptotic) from 1.94 ± 0.17% to 40.06 ± 1.12% (*p* < 0.001), while the viable cell population decreased correspondingly.

Collectively, these results suggest that acute MitoQ-induced mitochondrial perturbations—including excessive ROS production, collapse of oxidative phosphorylation, suppression of autophagic flux, and accumulation of dysfunctional mitochondria—ultimately converge on the activation of executioner caspases and apoptotic cell death in U87 cells. This positions apoptosis as a downstream effector of MitoQ-mediated mitochondrial failure and provides a mechanistic endpoint linking mitochondrial vulnerability to reduced cell viability.

## Discussion

GBM remains one of the most therapeutically refractory malignancies, largely due to its profound metabolic plasticity, mitochondrial adaptability, and intrinsic resistance to oxidative and genotoxic stress [22]. Increasing evidence indicates that ion channels—particularly KATP channels—serve not only as regulators of membrane excitability but also as integral modulators of mitochondrial bioenergetics and cellular stress responses in cancer cells [23]. Previous studies have highlighted the importance of potassium channel activity in shaping glioma proliferation, migration, and survival under metabolic stress conditions [7, 24]. However, the intersection between mitochondrial-targeted redox modulators and KATP channel infrastructure in GBM has remained largely unexplored. Based on the established sequence of mitochondrial dysfunction, our data are consistent with a model in which acute MitoQ exposure is associated with increased mitochondrial ROS production, followed by respiratory dysfunction, impaired autophagic flux, and apoptotic signaling. Importantly, these effects are accompanied by a selective downregulation of pore-forming and mitochondrial-associated KATP channel subunits, suggesting a previously unrecognized functional link between mitochondrial redox stress and KATP channel integrity in GBM.Collectively, these findings indicate that MitoQ rapidly induces a mitochondrial insult in U87 cells, characterized by severe superoxide accumulation. The extent of ROS generation—approaching that of a canonical complex III inhibitor— suggests that MitoQ overwhelms mitochondrial redox buffering capacity and bioenergetic stability. These observations suggest that mitochondrial ROS accumulation may represent an early event associated with subsequent bioenergetic impairment following MitoQ exposure.

Consistent with previous studies highlighting the role of potassium channels in cancer cell proliferation and survival [25], our baseline expression profiling revealed marked heterogeneity in KATP channel subunits across GBM cell lines. U87 cells displayed significantly elevated expression of both plasmalemmal (Kir6.2/SUR1) and mitochondrial-associated (CCDC51) KATP components compared to U251 and T98G cells. This finding is consistent with earlier reports demonstrating that ion channel expression patterns can stratify glioma subtypes based on proliferative capacity and metabolic phenotype [26, 27]. Importantly, our data identify a correlation between this transcriptional and translational enrichment and heightened vulnerability to mitochondrial perturbation in U87 cells. This finding generates the hypothesis that KATP channel expression levels may contribute to, or serve as a marker for, MitoQ sensitivity, though this requires direct functional validation.

In our GBM model, the primary mechanism of MitoQ action is consistent with that of a pro-oxidant mitochondrial disruptor, rather than an antioxidant. This aligns with a growing body of evidence demonstrating that in the high-stress environment of cancer cells, mitochondrial-targeted quinones can overwhelm endogenous redox defenses. As shown by Cheng et al., even a redox-crippled MitoQ analogue retains potent anti-proliferative activity against breast cancer cells, underscoring that mitochondrial accumulation and disruption of electron transport are central to its cytotoxic effect, independent of conventional antioxidant properties [16]. Our data confirm this, with MitoQ inducing mitochondrial superoxide accumulation comparable to the complex III inhibitor antimycin A. This redox disruption initiates a downstream cascade of dysfunction. The rapid and severe fragmentation of the mitochondrial network we observed is a hallmark of profound stress, leading to the loss of cristae integrity essential for oxidative phosphorylation. Consequently, we documented a dramatic suppression of respiratory parameters—basal and maximal respiration, ATP production, and spare capacity—signaling an irreversible bioenergetic catastrophe. This paradigm of pro-oxidant cytotoxicity is not unique to GBM but is corroborated by studies across various malignancies. In breast cancer, MitoQ has been shown to inhibit migration, invasion, and clonogenicity, effects linked to its ability to induce mitochondrial ROS and disrupt energy metabolism [18]. Similarly, in models of hepatic carcinogenesis, MitoQ demonstrated chemopreventive effects that were mediated through connexin- and p53-dependent pathways, again pointing to its role in modulating stress signaling and cellular homeostasis beyond mere antioxidant activity [20]. Research in colorectal cancer contexts has also revealed that MitoQ can suppress inflammasome-mediated cytokines, indicating its broad impact on inflammatory pathways often dysregulated in the tumor microenvironment [19]. Further reinforcing this broad-spectrum activity, a study in Ehrlich ascites carcinoma demonstrated that MitoQ combats tumor progression through a crosstalk involving elevated mitochondrial oxidative status, modulated mitophagy, and the suppression of NF-κB signaling [28]. The consistency of these findings—where MitoQ acts as a mitochondrial stressor—across breast, hepatic, colorectal, and now GBM models, strongly supports its classification as a broad-spectrum pro-oxidant anticancer agent whose efficacy is rooted in its capacity to destabilize the core metabolic organelle. An important consideration for interpreting studies with MitoQ is the potential contribution of its mitochondrial targeting moiety. MitoQ consists of a ubiquitone linked to a lipophilic triphenylphosphonium (TPP⁺) cation, which drives its accumulation within mitochondria. The TPP⁺ moiety itself can have biological effects at high concentrations. A limitation of the present study is the lack of a control compound consisting of the TPP⁺ targeting group without the active quinone (e.g., decylTPP). Such a control would help dissect the contribution of mitochondrial membrane potential-driven accumulation from the redox-active component of MitoQ. Future studies will incorporate this critical control to precisely attribute the observed cytotoxic effects to the quinone chemistry of MitoQ.

The structural consequences of this oxidative stress were evident in the profound fragmentation of the mitochondrial network observed following MitoQ treatment. In line with prior studies linking excessive ROS to suppression of mitochondrial fusion machinery and activation of fission pathways, U87 cells exhibited a dramatic reduction in mitochondrial length and branching, accompanied by a marked increase in fragmentation indices. Similar mitochondrial remodeling has been reported in cancer models exposed to mitochondrial toxins or redox disruptors and is often associated with impaired oxidative phosphorylation and increased susceptibility to apoptosis [29]. This is supported by ovarian cancer-specific research showing that altered mitochondrial dynamics, mediated by proteins like MFN2 and DRP1, are crucial for tumor cell survival and chemoresistance, making them a rational therapeutic target [30]. Notably, our data indicate that mitochondrial fragmentation occurred without a significant reduction in total mitochondrial mass, suggesting that MitoQ primarily disrupts network integrity rather than inducing immediate mitochondrial clearance. This observation aligns with findings in other malignancies; for instance, in breast cancer, MitoQ-induced mitochondrial fission has been directly linked to the loss of metastatic potential [18], and in prostate cancer, pharmacological induction of mitochondrial fragmentation promotes ROS-dependent cell death [31]. Thus, the morphological collapse we document in GBM cells is a critical and common component of a broader antitumor mechanism, where the disintegration of the mitochondrial network serves as a visible hallmark of irreversible metabolic crisis. This is further supported by research indicating that the antioxidant transcription factor Nrf2, often upregulated in cancer, can negatively regulate autophagy and growth arrest induced by MitoQ, suggesting that tumors with impaired Nrf2 signaling may be particularly vulnerable to this form of mitochondrial disruption [17].

A significant finding of this study is that MitoQ-induced mitochondrial stress is associated with the selective downregulation of pore-forming and mitochondrial-associated KATP channel components. While KATP channels are known to modulate mitochondrial membrane potential and ROS signaling in neuronal and cardiac models [2], their regulatory response within the context of oncogenic mitochondrial stress has been unclear. Our data show that the expression of Kir6.2 and CCDC51 is significantly reduced following MitoQ exposure. This observation raises the possibility of a feed-forward mechanism whereby initial mitochondrial dysfunction might impair the cell’s metabolic sensing apparatus, a hypothesis that warrants further investigation. Given that mitochondrial KATP channels contribute to maintaining organellar homeostasis and mitigating ROS-induced damage, their suppression could exacerbate mitochondrial instability and accelerate bioenergetic collapse [32]. This concept is supported by work in neurodegenerative models, where pharmacological modulation of KATP channels has been shown to influence mitochondrial dynamics, calcium handling, and cell survival [33]. Our finding that MitoQ downregulates Kir6.2 and CCDC51 connects to a growing interest in the role of ion channels in tumor biology. For example, in pancreatic ductal adenocarcinoma, KATP channel activity has been associated with metabolic reprogramming and chemoresistance [34]. Furthermore, studies in cancer models indicate that manipulating mitochondrial potassium flux can directly affect cellular proliferation and sensitivity to metabolic stress [35]. The selective downregulation of KATP channel subunits observed in our study may therefore represent the targeted disruption of a key adaptive mechanism, potentially leaving GBM cells less able to defend against MitoQ-induced metabolic attack. This aligns with the broader perspective that dysregulation of ion channels is a recognizable hallmark of cancer, contributing to tumor progression and therapy resistance, and thus represents a potential target for intervention [36]. Consequently, our data suggest that the mitochondrial KATP channel complex acts as a dynamic participant in the cellular stress response, and its expression levels may inform susceptibility to mitochondrial-targeted agents like MitoQ in GBM.

In addition to bioenergetic failure, our data reveal that MitoQ profoundly disrupts mitochondrial quality control by impairing autophagic flux. Although basal autophagic activity was preserved in untreated U87 cells, MitoQ-treated cells exhibited blunted LC3-II turnover and marked accumulation of p62, even in the presence of lysosomal inhibition. This pattern indicates a functional blockade of autophagic degradation rather than enhanced autophagosome formation. These findings are in agreement with prior work demonstrating that excessive ROS and mitochondrial depolarization can stall autophagic clearance, leading to the accumulation of dysfunctional mitochondria and further oxidative stress [37]. Importantly, defective autophagy has been increasingly recognized as a driver of tumor vulnerability, as it prevents the removal of damaged mitochondria that would otherwise sustain survival under stress. This is supported by studies in glioma and other solid tumors, where defects in mitophagy have been linked to increased sensitivity to chemotherapeutic agents and metabolic stress [38, 39]. The accumulation of p62 we observed may also have secondary consequences, as p62 can act as a signaling hub that activates pathways such as NF-κB, potentially contributing to the inflammatory tumor microenvironment [40]. The disruption of this critical quality control pathway by MitoQ therefore represents a key mechanism that exacerbates mitochondrial dysfunction and contributes to the overall cytotoxic outcome in GBM cells. While LC3-II and p62 report on general autophagic flux, their dysregulation in the context of profound mitochondrial damage is consistent with an impairment in the clearance of damaged organelles, including mitochondria.

Ultimately, the convergence of oxidative stress, mitochondrial fragmentation, bioenergetic collapse, KATP channel suppression, and autophagic failure culminated in the activation of executioner caspases and apoptotic cell death. The significant increase in caspase-3/7-positive U87 cells following acute MitoQ exposure confirms that these mitochondrial insults progress toward irreversible apoptotic commitment. This observation aligns with earlier reports demonstrating that MitoQ can activate intrinsic apoptotic pathways when mitochondrial stress exceeds a critical threshold in cancer models [16, 18]. The induction of apoptosis via mitochondrial dysfunction is a consistent therapeutic outcome observed with MitoQ across various cancers. In addition to breast cancer, studies in hepatic and colorectal carcinoma models have reported MitoQ-induced apoptosis linked to p53 activation and the suppression of survival pathways [19, 20]. Similarly, in Ehrlich ascites carcinoma, the antitumor effect of MitoQ was associated with the modulation of apoptotic regulators [28]. In GBM, the intrinsic apoptosis pathway is particularly relevant, as resistance to cell death is a major therapeutic hurdle [41]. The data presented here suggest that by delivering a potent, multi-faceted assault on mitochondrial integrity, MitoQ can overcome this resistance, forcing cells into an apoptotic program. This positions the activation of caspase-3/7 not merely as an endpoint, but as a definitive indicator of successful mitochondrial disruption and a promising indicator of therapeutic efficacy for mitochondrial-targeted strategies in GBM.

Several limitations of this study should be acknowledged. First, the mechanistic analyses were conducted primarily in the U87 cell line, which was selected due to its pronounced sensitivity to MitoQ. While the U87 cell line provided a robust and reproducible platform for this initial mechanistic dissection due to its pronounced MitoQ sensitivity, we recognize that this focus represents a limitation. GBM is characterized by profound inter- and intra-tumoral heterogeneity [22]. Therefore, the generalizability of these findings to other molecular subtypes, such as the more resistant U251 and T98G lines, remains to be established. The mechanisms operating in these less sensitive lines may differ fundamentally, and future studies must include a broader panel of patient-derived GBM cultures to validate the proposed mechanism. Second, it is crucial to emphasize that our data demonstrate only an association between KATP channel subunit expression and MitoQ sensitivity, not a direct mechanistic role. The observed downregulation, while suggestive, requires future functional validation using genetic knockdown or overexpression models, or specific pharmacological modulators of Kir6.2 and CCDC51, to establish a causal link to the observed mitochondrial failure and cytotoxicity. Third, the in vitro setting does not recapitulate critical aspects of the tumor microenvironment, such as hypoxia and stromal interactions, which could influence therapeutic efficacy [8]. Additionally, the primary viability screen relied on the CCK-8 assay. Future studies employing orthogonal methods such as clonogenic survival assays will be important to confirm the long-term cytotoxic effects of MitoQ across different GBM subtypes. Future studies should include functional validation using genetic knockdown or pharmacological modulation of specific KATP channel subunits to establish causality, and investigate the efficacy of MitoQ in patient-derived GBM cultures and orthotopic in vivo models to better assess its therapeutic potential. Therefore, validation in orthotopic in vivo models is essential. Furthermore, this study was designed to capture the early, initiating events of MitoQ action by focusing on a single 6-hour time point. While this approach successfully identified a cascade of acute mitochondrial perturbations, it does not provide a dynamic, kinetic understanding of how these events unfold over time. A detailed time-course analysis, mapping the sequence from ROS production to bioenergetic collapse and eventual apoptosis, is a necessary next step to fully delineate the temporal hierarchy of MitoQ’s cytotoxic mechanism. Fourth, a key limitation of this study is that the full mechanistic cascade—including mitochondrial ROS, fragmentation, bioenergetic failure, autophagic flux, and caspase activation—was only investigated in the U87 cell line. While U87 was deliberately chosen as the most sensitive model to establish a proof‑of‑mechanism, we have not yet confirmed whether similar pathways are engaged (or circumvented) in the more resistant U251 and T98G cells. Such validation is essential to determine whether the proposed mechanism is broadly applicable across GBM subtypes or is restricted to a particular metabolic phenotype. Fifth, mitochondrial ROS production was assessed using semi-quantitative fluorescence microscopy rather than fluorometric plate reader assays. While our approach provides single-cell resolution and was rigorously quantified, future studies should incorporate orthogonal methods such as flow cytometry or spectrophotometry to validate these findings. Sixth, this study did not include a non‑tumorigenic control cell line (e.g., HCN‑2 neurons or SVG p12 astrocytes) to evaluate the selectivity of MitoQ toward malignant cells. Such a control is critical for assessing the therapeutic window and potential off‑target toxicity of any candidate anticancer agent. We therefore acknowledge that the absence of these data precludes a definitive conclusion as to whether the cytotoxic concentrations identified in GBM cells are selectively effective or would also adversely affect normal neural cells. Seventh, while we demonstrate a clear impairment of global autophagic flux following MitoQ treatment, our experimental approach does not permit definitive conclusions regarding mitophagy specifically. The use of whole-cell lysates for LC3-II and p62 analysis, even in conjunction with bafilomycin A1, reports on the status of the general autophagy pathway but does not confirm whether damaged mitochondria are selectively engulfed by autophagosomes. Evaluation of mitophagy-specific markers, such as PINK1 stabilization, Parkin translocation, or co-localization of mitochondrial proteins with LC3-positive vesicles, is required to establish a direct link between MitoQ-induced mitochondrial damage and selective mitophagic clearance. Furthermore, the functional significance of the observed autophagic impairment—whether it acts as a failed protective mechanism or actively contributes to cell death—remains to be determined. Pharmacological modulation of autophagy (e.g., using Rapamycin or Torin 1) in combination with MitoQ, coupled with viability and caspase-3/7 readouts, would be necessary to address this question. These critical experiments are beyond the scope of the present two-phase mechanistic study, which focused on establishing the primary sequence of mitochondrial dysfunction. However, they represent a high-priority direction for our ongoing investigations and will be essential to fully delineate the role of autophagy in MitoQ’s anti-GBM activity.

**Fig. 8:**
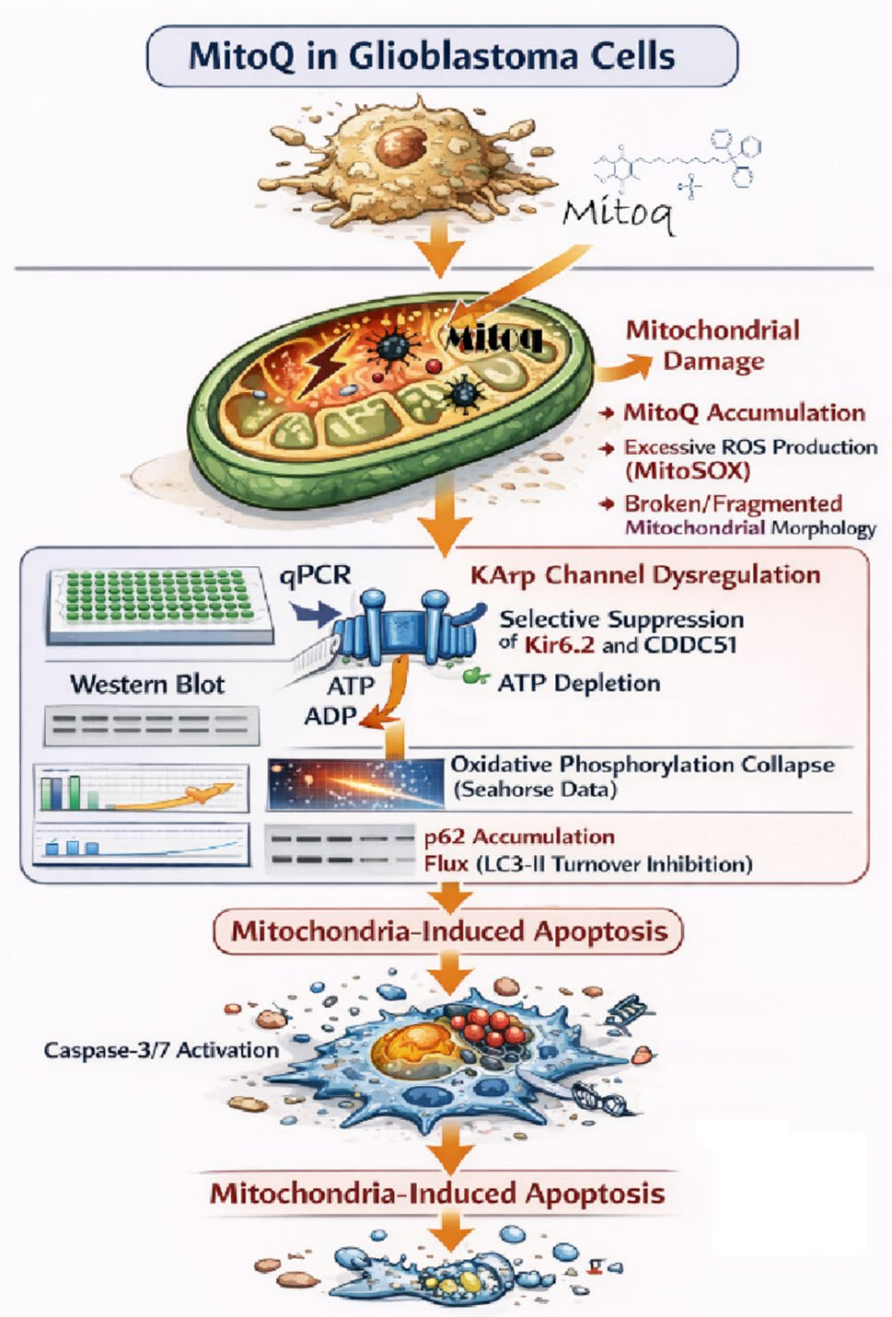
Schematic illustration of the proposed mechanism of MitoQ-induced mitochondrial collapse and apoptotic death in U87 cells

In conclusion, this study provides evidence that mitochondrial-targeted disruption by MitoQ exploits intrinsic metabolic vulnerabilities in GBM, with heightened effects in cells exhibiting enriched KATP channel infrastructure. By demonstrating a coordinated sequence of events—from pro-oxidant stress and KATP channel downregulation to mitochondrial fragmentation, bioenergetic failure, impaired autophagy, and ultimate apoptosis—our findings uncover a mechanistic axis of vulnerability in GBM cells. The integrated mechanistic pathway proposed in this study is summarized schematically in Fig. 8, illustrating the sequence from mitochondrial redox stress to KATP channel dysregulation, bioenergetic failure, impaired quality control, and eventual apoptosis. These results reinforce the concept that targeting core mitochondrial metabolism, rather than single oncogenic pathways, may offer a promising strategy to counteract the adaptive resistance that plagues current GBM therapies. While further validation in more complex models is necessary, this work underscores the potential of integrating mitochondrial disruptors like MitoQ with metabolic and ion channel-directed approaches in future neuro-oncology treatment paradigms. We acknowledge that the absence of mechanistic data in U251 and T98G cells represents a limitation, and that the therapeutic window of MitoQ relative to non‑malignant neural cells was not addressed in this study. This important question is now a central focus of our ongoing investigations, and we have outlined concrete plans to address this gap in subsequent work.

## Supplementary Information

Below is the link to the electronic supplementary material.

Supplementary Material 1:Assessment of nonlinear dose–response modeling.

Supplementary Material 2

## Data Availability

No datasets were generated or analysed during the current study.

## References

[1] Pardo LA, Stühmer W (2014) The roles of k(+) channels in cancer. Nat Rev Cancer 14:39–48. 10.1038/nrc3635 24336491 10.1038/nrc3635

[2] Tinker A, Aziz Q, Li Y, Specterman M (2018) Atp-sensitive potassium channels and their physiological and pathophysiological roles. Compr Physiol 8:1463–1511. 10.1002/cphy.c170048 30215858 10.1002/cphy.c170048

[3] Isomoto S, Kondo C, Yamada M et al (1996) A novel sulfonylurea receptor forms with Kir6.2 a smooth muscle type ATP-sensitive K⁺ channel. J Biol Chem 271:24321–24324. 10.1074/jbc.271.40.24321 8798681 10.1074/jbc.271.40.24321

[4] Foster MN, Coetzee WA (2016) Katp channels in the cardiovascular system. Physiol Rev 96:177–252. 10.1152/physrev.00003.2015 26660852 10.1152/physrev.00003.2015 PMC4698399

[5] Darwish R, Alcibahy Y, Abu-Sharia G, Butler AE (2025) β-cell mitochondrial dysfunction: underlying mechanisms and potential therapeutic strategies. Cells 14:1861. 10.3390/cells14231861 41369350 10.3390/cells14231861 PMC12691418

[6] Wawrzkiewicz-Jałowiecka A, Trybek P, Dworakowska B, Machura Ł (2020) Multifractal properties of BK channel currents in human glioblastoma cells. J Phys Chem B 124:2382–2391. 10.1021/acs.jpcb.0c00397 32129626 10.1021/acs.jpcb.0c00397 PMC7497650

[7] Maqoud F, Simone L, Tricarico D, Camerino GM, Antonacci M, Nicchia GP (2024) Functional interaction of Katp and BK channels with aquaporin-4 in U87 glioblastoma cells. Biomedicines 12:1891. 10.3390/biomedicines12081891 39200356 10.3390/biomedicines12081891 PMC11351575

[8] Aldape K, Brindle KM, Chesler L et al (2019) Challenges to curing primary brain tumours. Nat Rev Clin Oncol 16:509–520. 10.1038/s41571-019-0177-5 30733593 10.1038/s41571-019-0177-5 PMC6650350

[9] Du H, Xu T, Yu S, Wu S, Zhang J (2025) Mitochondrial metabolism and cancer therapeutic innovation. Signal Transduct Target Ther 10:245. 10.1038/s41392-025-02311-x 40754534 10.1038/s41392-025-02311-x PMC12319113

[10] Baharuddin WNA, Yusoff AAM (2025) Interplay of mitochondrial dysfunction and altered metabolic pathways in glioblastoma. Contemp Oncol 29:217–231. 10.5114/wo.2025.153848 10.5114/wo.2025.153848 PMC12518209 41098862

[11] Di Gregorio J, Petricca S, Iorio R, Toniato E, Flati V (2022) Mitochondrial and metabolic alterations in cancer cells. Eur J Cell Biol 101:151225. 10.1016/j.ejcb.2022.151225 35453093 10.1016/j.ejcb.2022.151225

[12] van den Bent MJ, Weller M, Wen PY, Kros JM, Aldape K, Chang S (2017) Clinical perspective on the 2016 WHO brain tumor classification. Neuro Oncol 19:614–624. 10.1093/neuonc/now277 28339700 10.1093/neuonc/now277 PMC5464438

[13] Yoshida GJ, Saya H (2021) Molecular pathology underlying the robustness of cancer stem cells. Regen Ther 17:38–50. 10.1016/j.reth.2021.02.002 33869685 10.1016/j.reth.2021.02.002 PMC8024885

[14] Kelso GF, Porteous CM, Coulter CV et al (2001) Selective targeting of a redox-active ubiquinone to mitochondria. J Biol Chem 276:4588–4596. 10.1074/jbc.M009093200 11092892 10.1074/jbc.M009093200

[15] Pang M, Wang S, Shi T, Chen J (2025) Overview of MitoQ in cardiometabolic diseases. Front Cardiovasc Med 12:1506460. 10.3389/fcvm.2025.1506460 40134978 10.3389/fcvm.2025.1506460 PMC11934253

[16] Cheng G, Karoui H, Hardy M, Kalyanaraman B (2023) Redox-crippled MitoQ inhibits breast cancer and glioma proliferation. Free Radic Biol Med 205:175–187. 10.1016/j.freeradbiomed.2023.06.009 37321281 10.1016/j.freeradbiomed.2023.06.009 PMC11129726

[17] Rao VA, Klein SR, Bonar SJ et al (2010) Nrf2 negatively regulates mitoquinone-induced autophagy. J Biol Chem 285:34447–34459. 10.1074/jbc.M110.133579 20805228 10.1074/jbc.M110.133579 PMC2966059

[18] Capeloa T, Krzystyniak J, d’Hose D et al (2022) MitoQ inhibits breast cancer migration and clonogenicity. Cancers 14:1516. 10.3390/cancers14061516 35326667 10.3390/cancers14061516 PMC8946220

[19] Dashdorj A, Jyothi KR, Lim S et al (2013) MitoQ suppresses NLRP3 inflammasome-mediated inflammation. BMC Med 11:178. 10.1186/1741-7015-11-178 23915129 10.1186/1741-7015-11-178 PMC3750576

[20] Qsee HS, Tambe PK, De S, Bharati S (2022) MitoQ mediates connexin- and p53-dependent chemoprevention. Toxicol Appl Pharmacol 453:116211. 10.1016/j.taap.2022.116211 36037915 10.1016/j.taap.2022.116211

[21] Kim S, Song J, Ernst P et al (2020) MitoQ regulates redox-related noncoding RNAs. Am J Physiol Heart Circ Physiol 318:H682–H695. 10.1152/ajpheart.00617.2019 32004065 10.1152/ajpheart.00617.2019 PMC7099446

[22] Bailleul J, Vlashi E (2023) Glioblastomas hijack metabolism for therapy resistance. Antioxid Redox Signal 39:957–979. 10.1089/ars.2022.0088 37022791 10.1089/ars.2022.0088 PMC10655009

[23] Leanza L, Checchetto V, Biasutto L et al (2019) Pharmacological modulation of mitochondrial ion channels. Br J Pharmacol 176:4258–4283. 10.1111/bph.14544 30440086 10.1111/bph.14544 PMC6887689

[24] Liu J, Qu C, Han C et al (2019) Potassium channels in glioma. Mol Membr Biol 35:76–85. 10.1080/09687688.2020.1729428 32067536 10.1080/09687688.2020.1729428

[25] Sesti F, Bortolami A, Kathera-Ibarra EF (2023) Non-conducting functions of potassium channels in cancer. Curr Top Membr 92:199–231. 10.1016/bs.ctm.2023.09.007 38007268 10.1016/bs.ctm.2023.09.007 PMC13135231

[26] Wang R, Gurguis CI, Gu W et al (2015) Ion channel gene expression predicts glioma survival. Sci Rep 5:11593. 10.1038/srep11593 26235283 10.1038/srep11593 PMC4522676

[27] Pollak J, Rai KG, Funk CC et al (2017) Ion channel expression in glioblastoma stem cells. PLoS ONE 12:e0172884. 10.1371/journal.pone.0172884 28264064 10.1371/journal.pone.0172884 PMC5338779

[28] Oraby MA, Elazazy O, Karam HM et al (2023) MitoQ modulates mitophagy and NF-κB signaling. Life Sci 331:122063. 10.1016/j.lfs.2023.122063 37666390 10.1016/j.lfs.2023.122063

[29] Comes N, Serrano-Albarrás A, Capera J et al (2015) Potassium channels in cancer progression. Biochim Biophys Acta 1848:2477–2492. 10.1016/j.bbamem.2014.12.008 25517985 10.1016/j.bbamem.2014.12.008

[30] Zou GP, Yu CX, Shi SL et al (2021) DRP1/MFN2-mediated mitochondrial dynamics in chemoresistance. J Cancer 12:7358–7373. 10.7150/jca.61379 35003356 10.7150/jca.61379 PMC8734405

[31] Baumgartner V, Schaer D, Moch H et al (2024) Mitochondrial elongation and ROS-mediated apoptosis. Int J Mol Sci 25:6939. 10.3390/ijms25136939 39000047 10.3390/ijms25136939 PMC11241170

[32] Shadel GS, Horvath TL (2015) Mitochondrial ROS signaling in homeostasis. Cell 163:560–569. 10.1016/j.cell.2015.10.001 26496603 10.1016/j.cell.2015.10.001 PMC4634671

[33] Evinova A, Okruhlica I, Racay P et al (2025) KATP channel modulation and mitochondrial dynamics. Neurochem Res 50:345. 10.1007/s11064-025-04598-2 41182570 10.1007/s11064-025-04598-2 PMC12583370

[34] Zhang J, Wang Y, Wang L et al (2024) Metabolic reprogramming in pancreatic cancer chemoresistance. Chin Med J 137:408–420. 10.1097/CM9.0000000000002758 37545027 10.1097/CM9.0000000000002758 PMC10876258

[35] Avolio R, Matassa DS, Criscuolo D et al (2020) Targeting mitochondrial metabolism to overcome chemoresistance. Biomolecules 10:135. 10.3390/biom10010135 31947673 10.3390/biom10010135 PMC7023176

[36] Prevarskaya N, Skryma R, Shuba Y (2018) Ion channels in cancer: are cancer hallmarks oncochannelopathies? Physiol Rev 98:559–621. 10.1152/physrev.00044.2016 29412049 10.1152/physrev.00044.2016

[37] Wang S, Long H, Hou L et al (2023) Mitophagy pathways in human disease. Signal Transduct Target Ther 8:304. 10.1038/s41392-023-01503-7 37582956 10.1038/s41392-023-01503-7 PMC10427715

[38] Caruso G, Laera R, Ferrarotto R et al (2024) Mitochondrial dysfunction in cerebral gliomas. Medicina 60:1888. 10.3390/medicina60111888 39597073 10.3390/medicina60111888 PMC11596904

[39] Li W, Xu X (2023) Mitophagy and mitochondrial apoptosis in glioblastoma. Front Pharmacol 14:1211719. 10.3389/fphar.2023.1211719 37456742 10.3389/fphar.2023.1211719 PMC10347406

[40] Yang X, Cao X, Zhu Q (2025) p62/SQSTM1 in cancer and DNA damage repair. Cancer Metastasis Rev 44:33. 10.1007/s10555-025-10250-w 39954143 10.1007/s10555-025-10250-w PMC11829845

[41] Karpel-Massler G, Ishida CT, Zhang Y et al (2017) Targeting intrinsic apoptosis in glioblastoma. Expert Opin Drug Discov 12:1031–1040. 10.1080/17460441.2017.1356286 28712306 10.1080/17460441.2017.1356286 PMC6143391

