# Supplementary figures and images for: MitoQ Triggers Mitochondrial Collapse and Apoptotic Death in Glioblastoma Associated with KATP Channel Expression Changes
Source: Neurochem Res. 2026 Mar 30;51(2):127. doi: 10.1007/s11064-026-04742-6 (PMC13035687; doi:10.1007/s11064-026-04742-6)

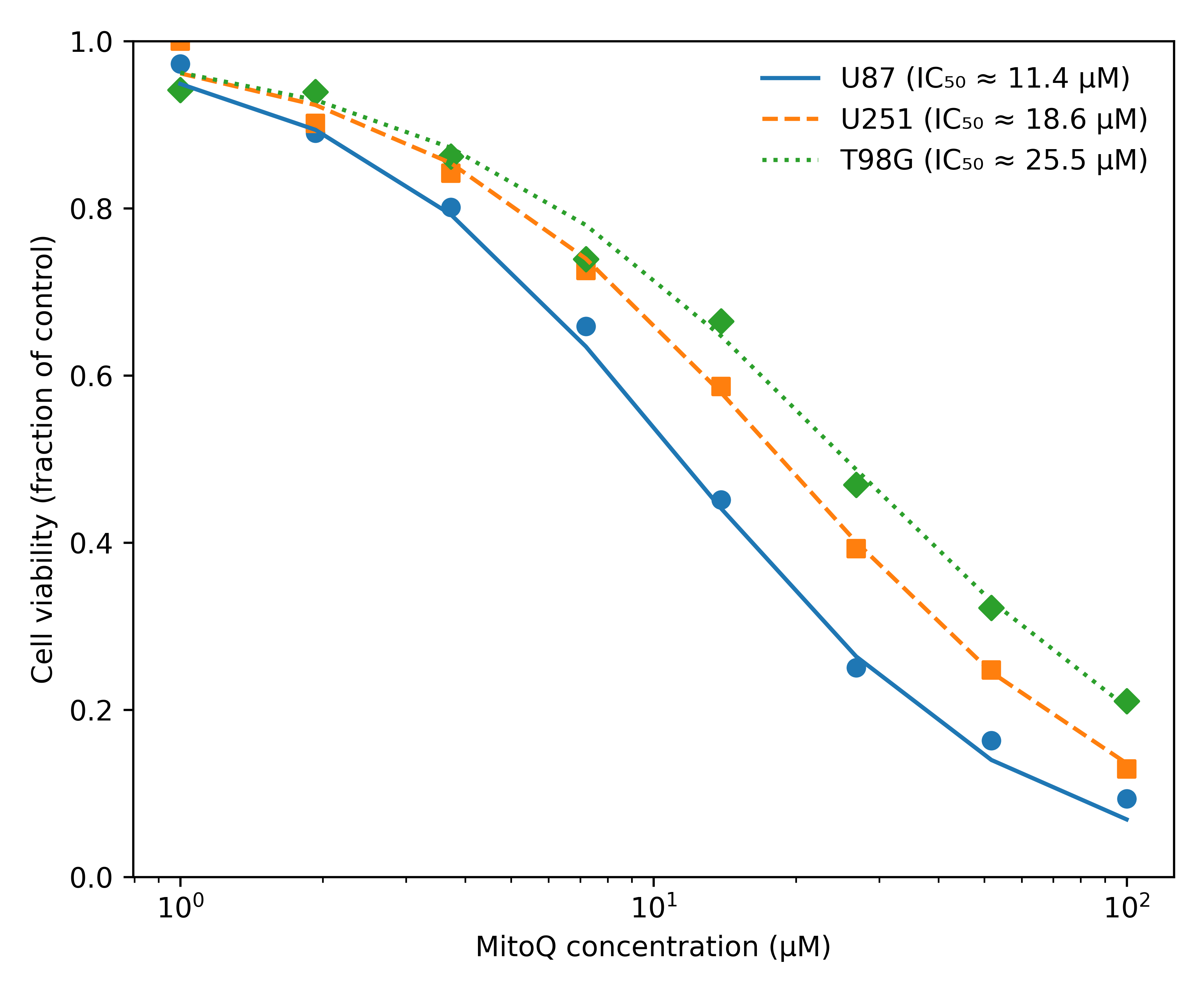

Supplement: Supplementary file 1 — Supplementary Material 1:Assessment of nonlinear dose–response modeling. [file 11064_2026_4742_MOESM1_ESM.png]
