# Supplementary material for: MitoQ Triggers Mitochondrial Collapse and Apoptotic Death in Glioblastoma Associated with KATP Channel Expression Changes
Source: Neurochem Res. 2026 Mar 30;51(2):127. doi: 10.1007/s11064-026-04742-6 (PMC13035687; doi:10.1007/s11064-026-04742-6)
Supplement: Supplementary file 2 — Supplementary Material 2 [file 11064_2026_4742_MOESM2_ESM.docx]

**Supplementary Table S1.** Quantitative analysis of MitoQ sensitivity and dose–response modeling across glioblastoma cell lines. Mean ± SEM values of IC₅₀ were derived from nonlinear sigmoidal regression of 24-h CCK-8 viability assays.

| **Cell line** | **IC₅₀ (µM)** | **R²** | **Statistical comparison** |
| --- | --- | --- | --- |
| U87 | 11.4 ± 0.8 | 0.9993 | p < 0.001 vs U251, T98G |
| U251 | 18.6 ± 1.1 | 0.9987 | p < 0.01 vs T98G |
| T98G | 25.5 ± 1.4 | 0.9989 | — |
